# Indigenous Service Provider Perspectives of an Online Education Module to Support Safe Clinical Encounters about Family Violence in Canada

**DOI:** 10.3390/ijerph192316061

**Published:** 2022-11-30

**Authors:** Christine Wekerle, Kahontiyoha Cynthia Denise McQueen, Bronwyn Barker, Anita Acai, Savanah Smith, Ilana Allice, Melissa Kimber

**Affiliations:** 1 Department of Pediatrics, McMaster University, Hamilton, ON L8S 4L8, Canada; 2 Optentia Research Unit, North-West University, Potchefstroom 2520, South Africa; 3 Offord Centre for Child Studies, McMaster University, BAHT 132, Hamilton, ON L8S 4L8, Canada; 4 Department of Psychiatry & Behavioural Neurosciences, McMaster University, Hamilton, ON L8N 3K7, Canada

**Keywords:** family violence, indigenous peoples, health professions education, trauma and violence-informed care, intimate partner violence

## Abstract

Given colonial genocide, Indigenous peoples are rightfully reticent to disclose their experiences of family violence to practitioners working within mainstream health care and social services. Health care and social service providers (HSSPs) have varied formal education on providing trauma-and-violence informed care to Indigenous and non-Indigenous families affected by family violence, including intimate partner violence and child maltreatment. The purpose of this study is to understand and describe the perspectives of Six Nations of the Grand River community members on the relevance of an education module to support HSSPs to provide physically and emotionally safe care to Indigenous families affected by family violence. Two-Eyed Seeing and Two Row Wampum approaches guided our qualitative study. Twenty-one (66.7% women) Indigenous HSSPs completed a semi-structured interview; 15 identified as a regulated HSSP, nine as a Knowledge Keeper/Cultural Holder, and three as a HSSP trainees. Conventional content analysis guided the development of codes and categories. The Violence, Evidence, Guidance, Action (VEGA)—Creating Safety education module was described as having elements consistent with Indigenous experiences and values, and supportive of Indigenous peoples seeking care from HSSPs for family violence related concerns. Participants described several suggestions to better adapt and align the module content with the diversity of values and beliefs of different Indigenous Nations. Collectively, the Creating Safety module may be used as an educational adjunct to Indigenous-focused, cultural safety training that can support HSSPs to provide physically, emotionally, and psychologically safe care to Indigenous peoples who have experienced family violence. Future work needs to consider the perspectives of other Indigenous communities and Nations.

## 1. Introduction

Family violence, which includes child maltreatment (CM) and intimate partner violence (IPV), is a globally pervasive problem with high costs to families and communities [1,2,3]. It has been routinely described as a serious public health and clinical service issue [4]. CM includes physical, emotional, sexual abuse and neglect of legal minors, as well as exposure to IPV between caregivers, causing, or is at risk of causing harm or injury to the child’s physical, psychological, and relational development [5]. IPV (also known as domestic violence) refers to physical, sexual, emotional, or financially abusive behavior by an intimate partner or ex-partner that can cause or causes physical, sexual, or psychological harm [6]. Over three decades of research in the field of family violence, which includes several systematic and meta-analytic reviews, has robustly linked exposure to family violence to the onset and persistence of negative health and well-being (e.g., [7,8,9,10,11,12]).

Given the health-related burdens of family violence, several public health authorities and family violence advocates have identified health care and social service providers (HSSPs) as having a key role in the prevention of family violence recurrence, as well as individual- and family-level sequalae. Yet reports indicate that HSSPs continue to receive inadequate training related to identifying and supporting people for whom family violence has been a concern. The impact of the COVID pandemic on risk factors for family violence has bolstered concerns related to adequately equipping HSSPs to be able to identify and respond safely to suspicions and disclosures of family violence in their practice encounters [13]. Specifically, public health protocols that were implemented to contain the impact and spread of COVID-19 (e.g., lockdowns), as well as the subsequent hardships related to virus outbreaks and public health measures (e.g., becoming ill, income/job loss, home-schooling) have been found to contribute to higher child abuse potential scores [14], new reports of IPV, and increasing levels of IPV severity [15]. In addition, dimensions of identity and axes of marginalization have been found to intersect and shape patterns of risk for family violence, its prevalence, and its impacts during and prior to the pandemic. For example, research suggests that people who are Indigenous to lands that are colonized are at increased risk of experiencing family violence [16,17] and Indigenous women in Canada reported greater concerns about the impact of COVID-19 for violence in their home compared to non-Indigenous women [18]. Adopting an intersectional perspective in the identification and response to family violence in health care and social service settings is therefore increasingly recognized as being critical for efficiently and effectively identifying and responding to family violence in health care and social service settings.

Intersectional perspectives conceptualize family violence as one of the many forms of gender-based violence that is shaped by interacting social norms rooted in white, individualist, ageist, and patriarchal vectors of colonial power and social control [19]. From this perspective, all forms of gender-based violence within Indigenous communities—including family violence—are considered a direct consequence of the colonial genocide and traumata of Indigenous peoples that has occurred since post-settler contact [20,21,22]. Specifically, colonial power and social control operationalized via racist policies and practices, such as land treaty violations, stealing Indigenous children for residential schooling, and coerced sterilization of Indigenous women, have directly disrupted (and in many cases, intentionally severed) historical, traditional, collective, and non-violent approaches to family, intimate partnerships, and child-rearing practices within Indigenous communities [6,23,24,25]. These racist practices and policies have been found to equally infiltrate health care and social service organizations and provider-patient interactions. For example, there is clear and compelling evidence that Indigenous peoples are disproportionately represented in reports to child welfare authorities and investigations of suspected maltreatment [25]. Indigenous peoples continue to experience racism in their encounters with health care and social service professions. This racism has had dire consequences, including death [26,27]. Within Canada, revived criticism of the harms that continue to occur for Indigenous peoples within health care and social service settings, the commitment to enact the United Nations Declaration on the Rights of Indigenous People [22], as well as political commitments to Truth and Reconciliation with Indigenous peoples, have together leveraged a plethora of efforts in HSSP training and educational efforts to improve the awareness of Indigenous-specific racism in health care encounters and mitigate its impacts [23]. An example of these efforts includes widespread training of HSSPs in trauma and violence-informed care.

Trauma and violence-informed care (TVIC) prioritizes safety in care interactions and settings to minimize the potential for re-victimization and the re-triggering of trauma symptoms in a way that is mindful of structural and systemic violence. This care is focused on strengths and resilience, and reinforces client agency in choice, collaboration, and connection. It is a policy stance of the Public Health Agency of Canada (see: https://www.canada.ca/en/public-health/services/publications/health-risks-safety/trauma-violence-informed-approaches-policy-practice.html (accessed on 5 May 2022)), and has been recently incorporated into the Creating Safety module within the Violence, Evidence, Guidance, Action—Family Violence Educational Resources (www.vegaproject.mcmaster.ca (accessed on 5 May 2022)), hereafter referred to as VEGA [28,29]. VEGA is a Canada-focused suite of freely available, web-based educational resources that were created to improve Canadian HSSP knowledge, attitudes, skills, and behaviors for safely recognizing and responding to family violence in their clinical encounters. Informed by the evidence indicating that HSSPs need more education related to family violence [30,31,32], the development of VEGA was led by Dr. Harriet MacMillan at McMaster University, funded by the Public Health Agency of Canada, and created in collaboration with 22 national-level stakeholder organizations whose representatives served on a National Guidance and Implementation Committee. These representatives included people representing the National Aboriginal Council of Midwives and the Canadian Indigenous Nurses Association [33]. Launched in 2020, VEGA encompasses four core modules that cover background information on family violence, concrete strategies for practicing TVIC, and practical guidance on how to recognize and respond safely to different forms of family violence, including CM and IPV [28,29].

VEGA’s TVIC module, entitled Creating Safety [29], introduces the ‘Environment, Approach, and Response (EAR)’ model as a conceptual and practical approach for attending to emotional and physical safety in health care encounters with people and families who are affected by family violence [29]. Although the Creating Safety [29] module was not developed as an ‘Indigenous Cultural Safety’ training, nor was it conceptualized as a viable replacement for cultural safety training more generally, the module’s curriculum and pedagogical strategies encourage HSSPs to think about and reflect upon (a) the multiple layers of trauma (e.g., historical and on-going) and violence (e.g., interpersonal, structural, systemic) disproportionately experienced by some communities, including Indigenous peoples, and (b) the relevance of that trauma and violence for safely identifying and responding to suspected or disclosed family violence in the service encounter. In addition, though not addressed in depth, the Creating Safety [29] module also discusses the ongoing impact of forced residential schooling and systemic racism for Indigenous peoples’ perceptions of safety in health care and social service settings, their comfort in accessing health care or social services for family violence concerns, and their possible reticence to disclose their family violence experiences [29]. Informal feedback from pre- and in-service professionals suggests that VEGA holds promise for improving HSSP knowledge, attitudes, skills, and behaviours related to safely recognizing and responding to family violence [34]. A formal evaluation of VEGA’s impact is currently underway [13]. However, a specific inquiry with Indigenous HSSPs related to the possible relevance of the Creating Safety [29] module to support culturally safe health care interactions with Indigenous families for whom family violence is a concern, has not yet been undertaken.

### Present Study

This study draws on evidence that culturally safe interactions with health care and social services facilitates client-centered care and has the potential to bolster the individual, family, and community resilience of Indigenous peoples to crisis and stress [35]. The research question guiding our work was, “how do Indigenous HSSPs working with members of the Six Nations (SN) of the Grand River community, describe the relevance and appropriateness of VEGA’s Creating Safety [29] module to support culturally safe approaches among HSSPs working with Indigenous individuals and families for whom family violence has been or could be, a concern?” More specifically, the specific objectives of the present study were to: (1) to describe SN HSSPs perceptions of the value and relevance of the Creating Safety module for HSSPs working with Indigenous clients; and (2) identify and describe adaptations needed to improve the module’s ability to support HSSPs to be able to provide culturally, physically, and emotionally safe clinical encounters with Indigenous clients.

## 2. Materials and Methods

### 2.1. The Setting

This study was led by the primary author, who is a White, non-Indigenous settler on the land her family refers to as ‘Canada;’ she led this work as a long-time collaborator with members of the Six Nations (SN) of the Grand River Community as an academic researcher employed by McMaster University. McMaster University is a research-intensive, post-secondary institution located in the city of Hamilton, Ontario, which is situated on the traditional territories of the Haudenosaunee, Anishinaabe, and Mississaugas of the Credit First Nations [36]. McMaster University neighbors the SN of the Grand River reserve, the largest Indigenous reserve in Canada, which is approximately 37.5 kms from the urban core of Hamilton. SN comprises less than 5% of the land originally granted to the SN community via the Haldimand Treaty of 1784 [37]. While the community continues to pursue land litigation with the provincial and federal governments to rectify several breaches of the original Treaty, the SN reserve is currently home to approximately 12,000 SN people, including peoples belonging to the Seneca (Onondowahgah), Cayuga (Guyohkohnyoh), Onondaga (Onundagaono), Oneida (Onayotekaono), Mohawk (Kanienjahagen), and Tuscarora (Ska-Ruh-Reh) nations [38].

### 2.2. Study Design

Approval for this study was received from the Hamilton Integrated Research Ethics Board, the Haudenosaunee Confederacy Council, and the SN Elected Council Research Ethics Committee. Permission to evaluate the Creating Safety [29] module for the present study was provided by McMaster University and Dr. Harriet McMillan (as the Lead of the VEGA Project) to the senior author (M.K.), as part of a larger research program evaluating the entirety of the VEGA Family Violence Education Resources among HSSPs across Canada [13]. As indicated in guidelines from the Tri-Council Policy Statement 2—Chapter 9 on Research Involving First Nations, Inuit, and Mètis Peoples of Canada [39], the procedures for the present study ensured written informed consent prior to data collection with participants, and consent was orally re-affirmed at the outset of our qualitative interviews (discussed below). Our qualitative work was informed by the principles of qualitative description [40,41], Two-Eyed Seeing [42,43] and Two Row Wampum [44] to honor the collaboration and inclusion of both Indigenous and non-Indigenous science, perspectives, and worldviews. Qualitative description is an applied health research methodology that is ideal for studies with an emphasis on generating knowledge and recommendations that have relevance for health care practitioners, educators, and policy makers [45]. As a form of naturalistic inquiry that was developed based on Western epistemology [41], it centers the perspectives and realities of a purposefully selected sample of participants who have in-depth knowledge and experience about a given health phenomenon; the centering of individual perspectives and experience is resonant with and respectful of Indigenous oral culture and storytelling. The methodology seeks to generate a factual and coherent synthesis of participant perspectives to position recommendations for further research to support health care practice, policy, and education.

To bring together Western and Indigenous science approaches in Canadian research, two concepts from Eastern Canada served as helpful guides. Specifically, Two-Eyed Seeing, and its sister principal Trees Holding Hands, were coined by Mi’kmaq Elder Albert Marshall to frame a co-learning journey, originally between Indigenous and non-Indigenous educators and researchers at Cape Breton University [46]. Trees Holding Hands arose from a teaching by a late Mi’kmaq Chief, Spiritual Leader, and Elder, Charles Labrador of the Acadia First Nation in Nova Scotia, who shared the following lesson with Todd, his son: “Go into the forest, you see the birch, maple, pine. Look underground and all those trees are holding hands. We as people must do the same” [46,47]. In 2004, Elder Marshall coined Two-Eyed Seeing to emphasize ‘us’ in knowledge sharing and knowledge generation, which he felt was not adequately expressed by the Trees Holding Hands principle [46,48]. Two Eyed Seeing refers to “Learning to see from one eye with the strengths of Indigenous knowledges and ways of knowing, and from the other eye with the strengths of Western knowledges and ways of knowing, and to using both these eyes together, for the benefit of all” [42].

In a similar vein, the Haudenosaunee Tekéni Teyohà:ke Kahswénhtake, or Two-Row Wampum, represents an approach to research knowledge exchange, within trusting relationships among equals, where the process or journey of the dialogical is significant [44]. There are three interlinking concepts: Kariwiio (good mind/equal justice), Kasastensera (strength in unity/respect), and Skenn:ne (peace) [42]. These concepts provide meaning and a basis for doing research in the SN Territory. As noted, it is important for researchers to be reflexive, and engage with their work in a way that is respectful to the cultural standards and protocols of the community. To this end, relationship-building between the first author (C.W.) and the SN community occurred in several ways. First, the primary author, who is a non-Indigenous academic researcher at McMaster University, partnered with a SN and McMaster University researcher, Dr. Dawn Martin-Hill, who was leading a holistic health research program centered on water issues (see: Global Water Futures [GWF] “Water is Life” or Ohneganos SN initiative: https://www.ohneganos.com/ (accessed on 5 May 2022)). As a co-investigator on the GWF project, the lead author of the present study developed a sub-grant that included the generation of the SN Youth Mental Wellness Committee, whose purpose is to advise on all research activities having relevance for youth resilience within the SN community. The committee was developed in 2019, and meets on a monthly or weekly basis. Consistent with the approach described by Freeman and Van Katwyk [44], these meetings began with visiting and food sharing before moving to the specifics of the research discussion, although meetings have subsequently moved to a virtual format following the onset of the COVID-19 pandemic. Relationship-building between CW and SN knowledge keeper Norma Jacobs (Elder-in-Residence, Wilfred-Laurier University, Waterloo, Ontario) also developed across regular meetings and followed the same principles as meetings with the SN Youth Mental Wellness Committee. Active allyship with SN was expressed through various opportunities for Western researchers and learners to attend and present at community information meetings, as well as to participate in community advocacy work and cultural events (e.g., powwow). 

This study’s protocols and procedures were informed by those previously established via the partnership described above, as well as the recommendations of the SN Youth Mental Wellness Committee. Specifically, this study was first presented (by M.K., C.W.) to the SN Health Services Leadership, and with their support, a meeting with the SN Youth Mental Wellness Committee was organized to discuss approaches for study recruitment. We then hired (a) an SN research assistant and project facilitator (K.C.D.M.), who is a Mohawk language speaker, and (b) two Elder advisors to guide research procedures. The SN Youth Mental Wellness Committee advised that approval for the research be sought via formal (a) submission of the research protocol for review by the SN Council Research Ethics Committee, and (b) presentation (by K.C.D.M. and C.W.) to the Haudenosaunee Confederacy Council via invitation to Council Meeting. With approval through both (a) and (b), the project protocol was submitted for research ethics review and approval by the Hamilton Integrated Research Ethics Board (Project #11565). Additional recommendations from the SN Youth Mental Wellness Committee and the team’s Elder advisors included focusing recruitment on SN resident HSSPs who worked with Indigenous peoples as their primary clients, patients, or mentees. In addition, a cash (or equivalent in retail gift card) honorarium for participation was recommended, as well as broadening the definition of HSSPs to be inclusive of people within the community who are Elders, Knowledge Keepers, and Cultural Leaders and who have a seminal role in community health and healing.

### 2.3. Researcher Reflexivity and Positionality

Researcher reflexivity and positionality are critical elements of promoting the trustworthiness of qualitative research and its findings. For Western academics conducting Indigenous-focused research, attending to, and acknowledging the ways in which one’s social location and identities influence the researcher’s perspective is a requisite. The first author (C.W.) is a White, non-Indigenous settler with parents who immigrated to Canada from Europe post-World War II. She has benefitted from Western education and financial awards in obtaining a Ph.D. (Clinical Psychology). While Dr. Wekerle’s work in CM stems from a child rights perspective, she has gained the Indigenous perspective of responsibility, rather than rights, in grateful relationship with and peacefulness among people, the attachment to and stewardship of the land, environment, and all beings in relationship, seen and unseen. She has collaborated with Indigenous child welfare leaders since the early 2000s and has engaged with Indigenous learners in her various publicly funded research projects. C.W. worked with a non-Indigenous staff member and learner on this project (S.S.), who was involved with the GWF initiative, attended the regular SN Youth Mental Wellness Committee meetings, obtained certification in Indigenous cultural safety, and completed the VEGA modules. Dr. Wekerle conducted all interviews with SN members, except for one participant, in accordance with a structured interview guide (see methods below).

Kahontiyoha Cynthia Denise McQueen (K.C.D.M.) was a research assistant on this project. She is Mohawk, Turtle Clan from SN Territory. Her academic background is Indigenous Studies and Global Health from McMaster University. She is a Mohawk language scholar and teaches the Mohawk language at SN’s Polytechnic. She is a frequent teacher of Indigenous culture and spirituality at various Ontario universities and colleges. As part of this project, Denise mentored the Western non-Indigenous research group in terms of Haudenosaunee culture, ways of knowing, and culturally safe research approaches. Denise assisted in the development of the interview guide and completed one of the semi-structured interviews (described below).

The last author (M.K.) and the research staff under her supervision (B.B., A.A., I.A.) are White and non-Indigenous, taking on self-study of Indigenous history, such as provided by the Woodlands Cultural Centre on SN, as well as completion of the San’yas Indigenous Cultural Safety Online Training Program (https://sanyas.ca/ (accessed on 5 May 2022)). Melissa is a registered and practicing social worker and psychotherapist, educator, and researcher of European (Scottish; English) settler ancestry. Her upbringing, education, and clinical practice has been dominated by Western and colonial frameworks. Since 2017, she has been a member of McMaster University’s Department of Psychiatry and Behavioural Neurosciences Indigenous Mental Health and Wellbeing Working Group. This is her first research project with the SN community; previous collaborations with Indigenous communities have focused on mental health service infrastructure within SN and the Mississaugas of the Credit First Nations reserves. She is recognized by Western colleagues as an expert in applied qualitative research methods and for the present study, oversaw the administration of research funds, the alignment of research processes with qualitative description, and the coding of data and integration of the advisory committee perspectives into the findings.

### 2.4. Sampling and Recruitment

A purposeful sample of HSSPs who met the following inclusion criteria were invited to participate in the present study: (a) working for one of 12 health care or social service organizations located on the SN reserve, serving on- and off-reserve members of the SN community (see Table 1); (b) at or over 18 years of age; (c) willing and able to complete the interview in English; and (d) willing and able to provide informed consent to participate [45]. We recruited an initial sample of participants by providing recruitment materials to directors and leadership of each of the organizations listed in Table 1 via email. Directors and leaders were asked to share the study information with their staff via internal listservs, as well as by posting the study information on notification boards throughout their agencies. We supplemented this approach with a snowball sampling strategy, whereby participants who completed data collection were asked to provide the names and contact information of others who they felt would offer important insight on the topic of this research study. Participants were also provided with the option to share information about the study with people in their personal and professional networks in order to respect privacy and mitigate undue pressure to participate.

### 2.5. Data Collection

Data were collected using semi-structured, one-on-one interviews with HSSPs [49,50]. The interview guide was initially drafted by Western members of the research team and collaboratively reviewed and revised by K.C.D.M.. The final interview guide explored participants’ general perspectives of VEGA’s Creating Safety module [29] and its content, definitions, and learning tools. Interviews also explored the key learning, knowledge, and practices needed amongst Indigenous and non-Indigenous HSSPs to support culturally safe interactions when working with Indigenous families who have experienced family violence. All participants completed one interview lasting between 45 and 75 min. The first interview was completed by the research assistant (K.C.D.M.), with remaining interviews completed by the first author (C.W.), due to time constraints. Participants could elect to complete their interview in person or virtually; except for one participant, whose interview took place in person on SN Territory, interviews were conducted over Zoom (Zoom Video Communications, Inc., San Jose, CA, USA). Data sufficiency for the present study was determined via the construct of information power, which informed our assessment of the sample size needed based on the research objectives, the homogeneity of the participants working within the community, and the quality of interview dialogue [51]. Interviews were completed between 25 August 2021, and 28 September 2021.

Review of our protocol by the appropriate committees (see Section 2.2 above) determined that our recruitment and data collection procedures posed minimal risk for participants. However, in recognition of the small chance that participants could experience anticipated or unanticipated thoughts or feelings related to the interview questions and the focus of the work on family violence, the interview team was trained to offer a resource list consisting of both community-specific and external support services, to participants disclosing any emotional distress during or at the conclusion of the interview. This resource list also included the names and contact information of members of the investigative team, including C.W. and M.K., the latter of whom is a registered and practicing social worker and psychotherapist in the province of Ontario and trained to provide immediate emotional support, where requested. 

### 2.6. Data Analysis

Data analysis was initially completed by a non-Indigenous research assistant (B.B.) under the supervision of the senior author (M.K.) by using an inductive approach to content analysis [52,53]. The research assistant read all transcripts in their entirety; she then conducted line-by-line coding of the full dataset and identified key concepts described by the research participants. Key concepts were clustered into discrete categories to describe participant perceptions of the module, its relevance for HSSPs working with SN community members who have experience with family violence, and any recommendations for improving the module’s ability to promote physically, emotionally, and culturally safe clinical encounters [29].

Throughout the analytical process, the research assistant extracted notable quotes to supplement and illustrate the descriptive summary of our data. Coding of the interview transcripts was managed through the program NVivo (QSR International, Burlington, MA, USA) [54]. To respect Indigenous traditions of group-based oral storytelling, learning, and knowledge generation [55], the lead author (C.W.) then provided the initial data summary to the SN Youth Mental Wellness Committee via oral discussion in a group-based virtual meeting; C.W. provided the overview of findings (as described below) and then listened to and hand-recorded the SN Mental Wellness Committee’s comments, reflections, and framing of those findings from their own perspective; we present that perspective in a separate section of the findings for the purposes of contextualizing and expanding the summary of the interviews, to respect the principles of qualitative description [40,41], Two-Eyed Seeing [42,43] and Two Row Wampum [44] approaches, and honor the inclusion of both Indigenous and non-Indigenous science, perspectives, and views in the interpretation of the findings. To this end, our descriptive summary of the coding process, as well as the narrative description offered by the SN committee, are presented as our results; these results are then integrated with the broader literature from both Indigenous and non-Indigenous authors in the discussion section.

## 3. Results

The interview findings include the perspective and experiences of 21 Indigenous HSSPs working directly with SN community members. Table 2 provides an overview of the sociodemographic characteristics of these participants. Greater than two-thirds of the participants (*n* = 14) self-identified as a woman, and just under half (*n* = 9) of participants indicated that they held role of a Cultural Leader, Knowledge Keeper, or Cultural Holder within the community. All participants self-identified as Indigenous; to protect the confidentiality of participants, we did not ask participants to self-disclose their specific Nation. When asked about the number of years they have been providing service to their community, four participants said they had been doing so “all of their life”, with one of these respondents indicating that their “responsibility began at birth, first to [my] parents [by] respecting them in how they lived their life”. The remainder of participants (*n* = 17) reported variable years in service to the community, with a range of 6 months to 30 years.

The qualitative interview data were organized within the following conceptual categories: (1) overall thoughts and opinions of the module; (2) potential adaptations to the module and perspectives on the core principles and definitions in the module; as well as (3) usefulness of each of the following elements of the module to support emotional and physical safety in the clinical encounter: (a) the TVIC visual guide, VEGA’s Environment, Approach, Response (EAR) Model; (b) case examples; and (c) the safe environment checklist [29].

### 3.1. Overall Thoughts and Opinions

There was consensus among participants about the overall value of the module to support HSSPs—both Indigenous and non-Indigenous—to be able to safely and effectively work with Indigenous peoples and families who experience family violence. Where Indigenous issues (e.g., residential schools) were discussed in the module specifically, this was seen as appropriate to HSSPs working with Indigenous individuals and families across Canada. Participants reflected that the module content and structure was unique, relative to other, previously encountered family violence education materials, because of its focus on prevention by better preparing HSSPs for clinical encounters with trauma histories.

What was important to HSSPs in clinical encounters with Indigenous families included: (1) physical, psychological, and spiritual safety that focuses on well-being, not only pathology and violence; (2) the explicit content supporting an intentional effort to establish and maintain trust with individuals and families; (3) respecting the role of the community in health and healing; and (4) ensuring that Indigenous and non-Indigenous practitioners have an authentic knowledge of past and ongoing cultural trauma that can influence historical or current experiences of family violence and its health impacts. Participants described the importance of recognizing that family violence is, in fact, a challenge among the community, but this challenge is complex, not insurmountable, and warrants a holistic, strengths-based approach. One participant captures this with the following:

*P11: I think this is really important. Like, being a cultural advisor, it’s like it’s not one–like I said, all of the departments that you talked about earlier, I have dealings with all of them, right? And a lot of them are, like some of the meetings are the same people, and they’re all in different departments so we hear a lot, I hear a lot of things, and it’s sort of like getting that realization of how bad it is, I guess, with our people. So, trying to turn things around and work on some positives. So, stuff like this really, really excites me because it’s happening, you know? We’re working towards something*.

None of the participants indicated disapproval or negative attitudes towards the module in its entirety, but where relevant, participants offered constructive critique. Critiques principally focused on elements of the module perceived to be very Western-centric. For example, participants described insufficient coverage of the importance of cultural traditions, connection to community, and connection to family/kinship/clanship as key facets of well-being among Indigenous peoples. For example, within the SN context, adolescent women are considered in the center of a circle of education about her partnering, with generations of integral relationships involving and revolving around women (i.e., mothers, sisters/cousins, Aunties, grandmothers). There is a deeply held, community-wide respect and responsibility for the safety and wellbeing of women and girls, and family more generally. This was not included in the module. Participants reflected how knowing about these traditional values and responsibilities and in some cases, the challenge or inability of being able to connect with those traditional values and responsibilities, influences how individuals and families experience family violence and what they do about it—which is relevant to the work of HSSPs with Indigenous individuals and families experiencing family violence. As one participant notes:

*P21: [The content is] very Western throughout… I think that, you know, just reinforcing back to culture and tradition, reaching out to your community, to make those connections, has been so beneficial. Understanding, at times, that we’re not necessarily able to do that, say we’re in a big city, limited services, things like that. But that’s the only piece that I would like to add into this, is that, you know, a lot of these individuals–what we think goes on is not necessarily what actually goes on. Multigenerational families living in households, individuals, you know, not necessarily being able to, if they’re in danger, to leave, and you know, we have this assumption just get up and go. Well, it doesn’t work that way for a lot of these people because they can’t leave their aunties, uncles, siblings, you know. So, understanding all of those pieces too, and how to work around that, to make sure that they’re having the adequate supports they need [is important].*

### 3.2. Possible Adaptations and Module Extensions

Suggested adaptions and extensions to the module focused on developing or recommending training for providers in cultural relevance, enhancing Indigenous-service provider’s safety, and deliberately expanding the training references to discuss structural violence.

With respect to developing or recommending training in cultural relevance, participants articulated the importance of provider understanding about the different ways in which Indigenous peoples live (e.g., on reserve, in urban settings, etc.), and the possibility for provider language and behavior to re-traumatize Indigenous peoples and families who have been exposed to cultural and structural trauma. Respondents suggested adding information to the module directly (or recommending/requiring external training) that improves Indigenous and non-Indigenous providers’ knowledge about the origins and impact of colonial actions, such as the Doctrine of Discovery, the Canadian Indian Act, the history of residential schools, the “Sixties Scoop”, and on-going trauma with discovery of child burials at residential schools. Extension of HSSP’s learning on the effects of these realities would enhance providers’ preparation for TVIC for Indigenous families who experience family violence. Captured by the quote, below, participants reflected that this information should include localized information to understand geographic differences so that HSSPs can appreciate the impacts of colonialism for the individuals and families that providers work with, to reduce the possibility of HSSPs experiencing helplessness and compassion fatigue, and to increase safety in the health and social service, across differing contexts:

*P04: When I have–when I meet people and I do talks, even students in my classes, you might learn all about what trauma-informed responses are, and you might know all of those things, but if you don’t know a fulsome history of the residential school system or the reservation system in Canada, and the history of the place, for example, that you work, that compassion fatigue will be compounded, because you will also start to–people start to question themselves about how they could not know, and what they could do, or have done about it, even though there’s nothing, there literally isn’t anything. So, sometimes the preventative is their own knowledge as well.*

Throughout the interviews, there was a consistent emphasis on the importance of appreciating and attending to ongoing traumas among Indigenous Peoples and the relevance of that trauma to experiences and discussions about family violence. Examples of these ongoing traumas include practices such as coerced sterilization, high out-of-pocket costs for basic needs (such as clean water), and failure among HSSPs and the public to acknowledge the genocide of Indigenous Peoples. These ongoing harms and their traumatic consequences were described as especially relevant to Indigenous HSSPs working with Indigenous individuals and families who are simultaneously experiencing these systemic traumas alongside experiences of family violence, which were noted to potentially exacerbate the risk of burnout and compassion fatigue among this much needed workforce. To that end, most participants made recommendations related to the protection and wellbeing of Indigenous HSSPs that they perceived could be readily integrated into the Creating Safety [29] module. Participants recommended that the module encourage providers and their organizations to seek out and make available culturally traditional supports, such as access to smudging rooms, community Elders, or traditional medicines. Five participants spoke about the need to explicitly acknowledge and consider the additional trauma that Indigenous HSSPs are carrying into the workplace, and the need to recognize and provide trauma-informed care, debriefing, and provider group cohesion and other supportive activities. As one participant explained:

*P20: I think too to note that often, as Indigenous providers, we are also seeking service, right? So, noting that, you know, the provider, like, is also the client often, right? In other areas. So, it’s not just vicarious trauma and compassion fatigue, but it’s also dealing with their own trauma, right? And I think that’s something to note.*

*Interviewer: Is there specific traumas for the Indigenous person that you would want to make sure are noted or mentioned or included?*

*P20: I think just noting that, you know, it’s not just being, like, vicarious trauma, like it’s not just about the client, it’s also that the provider is also dealing with their own trauma and their own, you know, trying to access supports and services, right? So, I think that’s something that is important, not necessarily anything in particular, because we’re all dealing with different things. But the reality is, you have people supporting people, but the people that are providing support are also being supported by the people, you know what I mean?*

### 3.3. Reflections on Core Principles and Definitions

Participants articulated suggestions for revising the module definitions and principles to align with Indigenous perspectives. Along with simplifying / shortening some definitions, these suggestions emphasized community, and acknowledging both ongoing and historical forms of violence toward Indigenous communities, rather than framing the trauma and violence as historical:

*P04: The difficulty is, right now, the forms of trauma are coming from [the discovery of residential school burial sites], but they’re very current as well. So, if anything, I would say current and historical.*

As an additional example, one participant noted that within the module, the term “genocide” was not used in the context of the genocidal violence experienced by Indigenous communities. Rather, the module used the term “genocide” in relation to violence that could be experienced by people or families who relocated to Canada as refugees. Participants recommended the use of the term “genocide of Indigenous peoples” and advised against the use of alternate terms, such as “cultural genocide”. 

### 3.4. Utility of VEGA’s EAR Model, Case Examples, and Safe Environment Checklist for Working with Indigenous Communities

Informed by the principles of TVIC, the intention of VEGA’s Environment, Approach, Response (EAR) Model [29] is to provide conceptual and action-oriented strategies for HSSPs to create safe environments and interactions when working with people and families who have experienced family violence. In reviewing the EAR model and associated content, participants in the study focused their commentary on its relevance (or lack thereof) for ensuring Indigenous patient safety, suggestions for improving the model’s applicability to environments and interactions that include members of Indigenous communities, as well as more general reflections about its appeal and utility.

Participants unanimously affirmed the relevance of the VEGA EAR model [29] for working with Indigenous communities affected by family violence. Participants reflected on the practical utility of each element of the model (“E” or Environment, “A” or Approach, and “R” for Response) [29], with its three sections and associated clinical tips. Participants described the helpfulness of the model graphic, which helped to orient providers to the importance of attending to safety in the process of the interaction, the content of the interaction, and the setting of the interaction. These sentiments are reflected by one participant, in the following:

*P07: Ah, I think that the visual is fantastic. I remember when I first did this module, I was like, ‘Oh, this would be so good to bring into a staff meeting!’ Like, I hope that people could learn from something like this because it’s really easy to, like it’s an easy graphic to look at. And it also provides really simple, straightforward tips. I think that incorporating this sort of practice into organizational level of care would benefit many more clients if people adhered to something like this. And it seems quite simple, like it doesn’t even, like, ask for organizations or social workers or counsellors to go that far outside of their regular practice.*

With respect to improvements to the model, participants offered a variety of suggestions, including providing examples of how to approach an Indigenous person and start a conversation regarding family violence. Participants noted that this is especially important given that some provider-patient interactions may be within the context of a home visit or a different environment that the provider cannot directly control. Participants reflected that these possibilities emphasize the responsibility of the provider to educate themselves on the preferences of service provision for local Indigenous communities to avoid a pan-Indigenous approach to caring for families affected by family violence.

Eight of the 21 participants offered general reflections about the color palette of the VEGA EAR model [29], its associated graphics, and the module, more generally. Three of these respondents articulated their appreciation for the alignment between the color palette (white and purple) with the Ayenwahtha wampum belt, which documents the establishment of the Haudenosaunee Confederacy. On the contrary, the remaining five participants cautioned against using colors representative of a specific nation, with one participant suggesting that it be made clear that the colors used by VEGA were selected by the VEGA Project Team [28], and therefore, any color alignment with any specific Indigenous community or communities is coincidental.

Eighteen of the participants described appreciation for case examples that did not reinforce negative stereotypes about Indigenous peoples and families. A small number of participants also offered amendments to the case scenarios to better align with how and when patient information is typically made available within the clinical interaction, and pragmatic opportunities to inquire about family violence given certain client information. For example, one participant offered the following:

*P17: I just wonder if the ordering of the case itself could be improved, because it’s like it’s giving you all this information, past information right away, but you don’t always know that […] if someone’s just coming in for a flu shot, right? Unless you’re having a longer conversation with them.*

Lastly, participants unanimously supported the availability and components of the “safe environment checklist [29]”, which details a list of eight suggestions or considerations for providers to improve the emotional and physical safety of their workplace. Two-thirds of the participants (*n* = 14) also volunteered additional considerations for a revised list, some of which included: signage in local Indigenous languages; wall art by Indigenous artists who are local to the practice setting; asking the clients or service-users what makes a space safe for them; asking for feedback on the space; expanding the accessibility of the space (e.g., considering hours of operation, physical accessibility for people with assistive devices or strollers); prioritizing a strengths-based approach to addressing whatever presenting concern is raised by the client/patient; and providing access to culturally specific resources, such as community Elders or a space for cultural practices such as smudging or burning tobacco. Participants thoughtfully connected these practical considerations to key elements of Indigenous health and wellbeing, such as connecting with the community:

*P20: I think [the list is] good. Because this is an overarching training, right? It’s more sort of generalized to any service provider. I think it’s good […] even for us, not just displaying welcome signs in local languages, but is the art on our wall local artists, right? So, that’s something that we even think about for our space, like are we including local Indigenous artists? Is that who’s on our walls, right? Is part of our community on our walls? So, I think that that’s something that we consider. But I think that’s a good checklist.*

### 3.5. SN Youth Wellness Committee Reflections

In reflecting on the analysis summary with the primary author, Committee Members reinforced the importance of ensuring that the module acknowledge there is no single pan-Indigenous approach to recognizing and responding to family violence. Different communities, families, and people have had various traumatic experiences that do and do not include family violence, and each Indigenous community and Nation has been (and continues to be) differentially affected by ongoing and historical forms of trauma against Indigenous peoples. In sharing these perspectives, Committee Members reflected on the recurring discovery of unmarked graves and burial sites at Canada’s former residential schools, the need for Indigenous peoples to continue to grapple with the impacts of racism related to Canada’s genocide of Indigenous peoples, as well as “survivor’s guilt” for those who (or whose ancestors) survived or escaped the residential school experience.

In reflecting on the analysis summary, Committee Members identified a need for the module to provide information or further links to regionally specific Indigenous communities and Nations. Committee Members discussed the importance of making localized knowledge available to ensure that HSSP recognition and response to family violence is respectful and attentive to the experiences of Indigenous clients and communities that are most likely to access their care. Committee Members discussed and reflected on the importance of providers understanding intergenerational trauma and the daily, less overt impacts of the residential schooling system on survivors and their families. In considering the findings from the interviews, Committee Members offered additional examples of these effects, including lack of trust in authority figures, poor self-care practices, low willingness to seek services, and nutrition-related challenges. These effects were perceived to be less well known or discussed, but equally important to a holistic perspective of health, wellbeing, and the impacts of colonial trauma.

Consistent with interview participants, Committee Members suggested revisions to key terms and definitions within the module. In particular, the term “cultural humility” was uniformly proposed as a more appropriate alternative to the module’s use of the term cultural competence, cultural knowledge, or cultural safety. Members discussed that these are Western and clinical terms that may not actually be safe for all Indigenous peoples. They positively reflected on the perspective of one interview participant who indicated that cultural humility offers room for growth in knowledge and acknowledges that no person is completely competent in any culture, even their own [56]. Simply put, cultural humility allows one to be humbled by the reciprocity of relationships. Committee Members expressed caution regarding the expectation that all Indigenous people in a community will have the same level of knowledge or understanding of their culture. In parallel, they articulated the importance of reducing the possibility of divisiveness among community members via language and practices that could foster shame (e.g., “who is a better Native”), especially among, for example, some residential school survivors, where the possibility to embrace traditional languages and practices was stolen from them or invoked severe and recurring punishment.

The conversation about definitions and concepts also considered the use of “peace” instead of safety, as a term that is more resonant to and valued by their community. Committee Members described that promoting a safe (or peaceful) environment includes, and goes beyond, displaying artwork of local Indigenous artists and attending to the physical space of the agency; they described it instead as walking side-by-side with clients to demonstrate acceptance and value. It was noted that, within Indigenous communities and service organizations, lateral violence may be present. Lateral violence—also referred to as internalized colonialism—recognizes the power-based dynamics arising from the internalization of values and aggressive behaviors of oppressors that represent an abuse of power. It is considered to develop from patriarchal society governance and development, as Indigenous societies are matriarchies historically [57]. Examples offered by Committee Members included the potential for outside-of-work disputes to be carried into workplace relationships, and privacy disruptions during help-seeking, given visibility and knowledge of resident’s vehicles. Committee Members re-affirmed the need for dedicated reflection spaces, access to Elders, opportunities for work group socialization, and ongoing training opportunities for all staff members in traditional teachings, such as the Haudenosaunee Great Law of Peace. Relatedly, the group raised concerns surrounding discussing violence in too small of a setting, seeing this as potentially threatening, and suggested the opportunity for natural activity settings (e.g., Nature walk) as opposed to an office or a meeting room. As was discussed in the participant interviews, attending to an Elder’s safety and personal history is a relevant and important consideration for HSSPs, as Elders may have their own history of family violence, or know or be a relative of a person who uses violence in their relationships or is being victimized.

Lastly, Committee Members spent considerable time discussing and reflecting on the term “Elder”. They indicated that not all Indigenous Nations and communities use the term “Elder”, as its meaning (and the role) may not be clear. Communities may prefer the term “Knowledge Keeper” “Faith Keeper”, or “Cultural Advisor”, which is important for respectfully ensuring relevance of the module content for all Indigenous audiences. From the Committee’s perspective, this consideration reiterated the need to encourage HSSPs to intentionally learn about local Indigenous communities to respectfully engage with their clients and patients about the possible support that an Elder, Knowledge or Faith Keepers or Cultural Advisor can offer their family in the healing process. Committee Members stressed that if a provider or clinic is requesting the services of an Elder or prominent community member in a similar capacity, it is important to be open-minded and reflect upon the Elder’s input to apply their teachings respectfully and effectively.

## 4. Discussion

This study explored Indigenous HSSPs’ perspectives regarding the value and utility of the Creating Safety module of the Violence, Evidence, Guidance, Action—Family Violence Education Resources [29] to support HSSPs’ knowledge, attitudes, skills, and behaviors related to safely recognizing and responding to suspected or disclosed family violence among Indigenous families. Overall, the module was received positively by the research participants. More specifically, its use of visual aids, and applied case examples were enthusiastically received. Participants also offered several recommendations for improvements for the module, including increasing the information provided about the physical safety of HSSPs’ workplaces and deepening the attention to the emotional safety of Indigenous HSSPs, who may also be living with family violence. While Indigenous HSSPs reported being able to “see” themselves in the case content that was not specific to Indigeneity, participants described Indigenous-specific suggestions for the module which could be supportive of cultural safety in the health care or social service encounter, including making services available in Indigenous languages, posting art by Indigenous artists, and facilitating access to culturally specific supports and spaces.

Additional recommendations for the Creating Safety [29] module included providing more content related to the scope of violence experienced by Indigenous communities and the multigenerational impacts of that violence. Participants described these recommendations alongside suggestions to link module users to further learning about colonial atrocities, including residential schooling, the Sixties Scoop, and the disproportionate murder and abducting of Indigenous women and girls, among others. Participants were consistent in discouraging a pan-Indigenous approach to providing health care and social services to Indigenous families affected by family violence and emphasized that the localization of content is essential to support specific Nations and their geographical contexts.

Participant recommendations can be framed within the action imperative for Truth and Reconciliation efforts [21]. While academic and clinical institutions have nearly uniformly adopted land acknowledgements, research indicates that these statements have become viewed by many Indigenous peoples as performative, and rarely coincide with the personal research, historical truth understanding, restorative action and outreach recommended for true reconciliation efforts [58]. Relatedly, participants were clear that the information offered by the Creating Safety [29] module was not enough to ensure physical and emotional safety in the context of service provision to Indigenous families who have experienced family violence. Participants centered the need for HSSPs to absorb themselves in learning about the historical and ongoing ways in which colonialism impacts local Indigenous communities. This included learning about the ways in which one’s professional identity, a HSSP’s respective organization, and their broader community have participated in and reinforced colonial ideology, practices, and harms toward Indigenous communities. Resonating with the findings of a scoping review completed by our team and which collated the international literature on cultural safety in family violence service provision [59], participants encouraged critical reflexivity about the impact of these ideologies and practices in the onset of family violence in Indigenous communities, how the pervasiveness of family violence is maintained in Indigenous communities, and why connection to culture and community are so central to healing.

There are some limitations of this study. First, perspectives come solely from Indigenous HSSPs serving the SN of the Grand River community, representing the Haudenosaunee Peoples of the Longhouse. Further research of the Creating Safety module of VEGA’s Family Violence Educational Resources [29] is needed with other Indigenous communities. Second, this study involved participants who volunteered to take part, and therefore, may have been more interested in and knowledgeable about family violence. We did not ask participants whether they had personal experiences with family violence. It is also possible, therefore, that given the findings about the possibility of intra-community awareness and stigma influencing family violence help-seeking and disclosure, that those with experiences of family violence did not feel comfortable participating. One participant suggested a longer amount of interview time, or that multiple interview sessions may be fruitful in further research to collate more examples of how to integrate Indigenous-focused content. There were considerably more women participants than men, and no one in the sample identified with a gender identity other than woman or man. Greater participation of women in our study may reflect their greater propensity to participate in interview-based studies, as well as the greater proportion of women that tend to work in the mental health and family violence service fields [60]. Finally, we acknowledge that only one member of the research team self-identifies as Indigenous; this member of the team is also (from a Western science perspective) an early-career researcher. The imbalance with respect to the number and career stage of Indigenous researchers on the team could have had bearing on the interpretation of the findings presented herein. However, it is our hope that the intentional and prolonged consultation with the Six Nations Youth Mental Wellness Committee and the explicit presentation of their perspectives in the findings assists in tempering any perceived or actual imbalance. In addition, it is important to note that reflections offered by members of the Six Nations Youth Mental Wellness Committee were comprehensive and captured in a group-based format; it is possible that the nature of group-based versus one-on-one consultation with group members could have bearing on the nature and extent of reflections offered.

## 5. Conclusions

The VEGA Family Violence Education Resources [29] are freely available in both English and French and present as an important educational opportunity for Indigenous and non-Indigenous HSSPs to improve their knowledge, attitudes, skills, and behaviors related to recognizing and responding to family violence in their service encounters. The Creating Safety [29] module incorporates key principles of TVIC to support HSSPs to work with Indigenous and non-Indigenous families affected by family violence in an emotionally, physically, and culturally safe manner. Cultural safety has been identified as paramount for Indigenous peoples when seeking care from non-Indigenous centers that are primarily staffed with non-Indigenous professionals. A lack of trust, visible safety signs, and a felt sense of security has been identified to contribute to Indigenous peoples’ reticence to access health care and social services, as well as disclose their health concerns, including family violence [61,62]. While our data were collected in the context of participants review of VEGA’s Creating Safety module, our research approach and our findings are likely applicable to the evaluation and implementation of other educational interventions focused on family violence, as well as health care and social services more generally. Participants noted several important elements of the Creating Safety [29] module for strengthening, including: greater emphasis on the importance of cultural traditions, as well as community, family, and kinship connection on individual health and healing for Indigenous people following family violence; increased attention on the continuation of structural violence toward Indigenous communities; the explicit recommendation for HSSPs to undergo cultural safety and/or relevance training; as well as, specific strategies for enhancing Indigenous HSSPs’ emotional and physical safety at work. In general, however, the overall perspectives of Indigenous HSSPs towards the VEGA’s Creating Safety module [29] for improving emotional, physical, and cultural safety in clinical encounters where family violence is a concern, are positive, and this suggests that the module may be a fruitful educational adjunct to Indigenous-focused, cultural safety training that can support HSSPs to provide physically, emotionally, and psychologically safe family violence services that are rooted in the cultural practices of particular Nations.

## Figures and Tables

**Table 1:** Health care and Social Service Agencies Involved in Recruitment.

Organization/Agency Name
Six Nations Health Services
Ganǫhkwásra Family Assault Support Services
Tsi Non:we Ionnakeratstha Ona:grahsta’ (Six Nations Birthing Centre)
Egowadiyadagenha’ Land-Based Healing Centre/Traditional Medicine Clinic
Six Nations Emergency First Response Team
Six Nations Family Health Team
Six Nations Police Service
Six Nations Health Services Mental Health Team
Gane Yohs Health Centre—Sexual and Clinical Health Nurses
Ogwadeni:deo Child Protection Services
Kanikonriio (Good Mind) Youth Life Promotion Program—Aboriginal Children’s Mental Health and Addiction Program
Six Nations Health Services Health Promotion and Nutrition Services

**Table 2:** Sociodemographic Characteristics of Research Participants.

Variable	Category	Frequency (%)
Community Role ^a^		
	Knowledge Keeper/Cultural Holder	9 (43)
	Regulated Health care or Social Service Professional	15 (71)
	Trainee	3 (14)
Gender		
	Woman	14 (67)
	Man	7 (33)
	Two Spirit	0 (0)
Age ^b^		
	18–25 years	2 (10)
	26–30 years	2 (10)
	30–35 years	3 (14)
	36–40 years	1 (5)
	41–45 years	6 (29)
	46–50 years	3 (14)
	50 years or greater	4 (19)
Age Demographic for Services ^a^		
	Children	3 (14)
	Adolescents	5 (24)
	Adults	3 (14)
	Families	0 (0)
	All of the Above	16 (76)

Table Notes: ^a^ Values do not add to 100% given the opportunity to select more than one relevant option. ^b^ Values do not add to 100% due to rounding.

## Data Availability

This data set is not available for sharing outside of the research team. When participants consented to participating in the study, they did not consent to access to transcripts or data beyond the research team. Some complete qualitative transcripts may be identifiable. Please contact the corresponding author for further clarification.
